# Polymorphisms of Pro-Inflammatory IL-6 and IL-1β Cytokines in Ascending Aortic Aneurysms as Genetic Modifiers and Predictive and Prognostic Biomarkers

**DOI:** 10.3390/biom11070943

**Published:** 2021-06-25

**Authors:** Letizia Scola, Rosa Maria Giarratana, Vincenzo Marinello, Valeria Cancila, Calogera Pisano, Giovanni Ruvolo, Giacomo Frati, Domenico Lio, Carmela Rita Balistreri

**Affiliations:** 1 Clinical Pathology, Department of Bio-Medicine, Neuroscience, and Advanced Diagnostics, University of Palermo, 90100 Palermo, Italyrosamaria.giarratana@unipa.it (R.M.G.); carmelarita.balistreri@unipa.it (C.R.B.); 2 Department of Legal and Economic Sciences, University of Enna “Kore”, 94100 Enna, Italy; vincenzo.marinello@unikore.it; 3 Tumor Immunology Unit, Department PROMISE, University of Palermo, 90100 Palermo, Italy; valeria.cancila@unipa.it; 4 Department of Cardiac Surgery, University of Rome ‘Tor Vergata’, 00100 Rome, Italy; calogera.pisano@uniroma2.it (C.P.); giovanni.ruvolo@uniroma2.it (G.R.); 5 Department of Medical-Surgical Sciences and Biotechnologies, Sapienza University of Rome, 04100 Latina, Italy; giacomo.frati@uniroma1.it; 6 IRCCS NEUROMED, 86077 Pozzilli, Italy

**Keywords:** thoracic ascending aortic aneurysms, proinflammatory cytokines, rs1800795, rs16944, telomere length, MMP9, elastic fragmentation, medial cell apoptosis, cystic medial changes

## Abstract

Background: Previous studies have demonstrated that polymorphisms involved in immune genes can affect the risk, pathogenesis, and outcome of thoracic ascending aortic aneurysms (TAAA). Here, we explored the potential associations of five functional promoter polymorphisms in *interleukin-6* (*IL-6*), *IL-1B, IL-1A, IL-18, and Tumor necrosis factor (TNF)A* genes with TAAA. Methods: 144 TAAA patients and 150 age/gender matched controls were typed using KASPar assays. Effects on telomere length and levels of TAAA related histopathological and serological markers were analyzed. Results: Significant associations with TAAA risk were obtained for *IL-6* rs1800795G>C and *IL-1B* rs16944C>T SNPs. In addition, the combined rs1800795C/rs16944T genotype showed a synergic effect on TAAA pathogenesis and outcome. The combined rs1800795C/rs16944T genotype was significantly associated with: (a) higher serum levels of both cytokines and MMP-9 and -2; (b) a significant CD3+CD4+CD8+ CD68+CD20+ cell infiltration in aorta aneurysm tissues; (c) a significant shorter telomere length and alterations in telomerase activity. Finally, it significantly correlated with TAAA aorta tissue alterations, including elastic fragmentation, medial cell apoptosis, cystic medial changes, and MMP-9 levels. Conclusions: the combined rs1800795C/rs16944T genotype appears to modulate TAAA risk, pathogenesis, and outcome, and consequently can represent a potential predictive and prognostic TAAA biomarker for individual management, implementation of innovative treatments, and selection of the more proper surgical timing and approaches.

## 1. Introduction

In recent decades, inflammation has been demonstrated to have a crucial role in cardiovascular diseases (CVD) [1,2], characterized by high worldwide morbidity and mortality. Accordingly, inflammation evocates endothelial dysfunction, the primum movens of many CVD, from the atherosclerosis [3,4] and its complications (i.e., myocardial infarction, coronary artery disease, ischemic stroke, peripheral arterial occlusive disease, and heart failure [2,4,5,6]), to other cardiovascular pathological conditions [7]. In addition, individuals affected by chronic inflammatory disorders (i.e., autoimmune disorders) show a significant increase in the cardiovascular susceptibility. [6] Many inflammatory pathways have been documented to contribute to CVD onset and progression, including TLR-4/NF-kβ pathway [8,9], TLR-2 [10], TGF-β1 [11], CCR5 [12], CRP [13]. Recently, pro- and anti-inflammatory cytokines, such as interleukin-(IL)-10 [14] and IL-6 [15], have been associated with CVD onset and progression, and current meta-analyses [16,17,18,19] have also confirmed the relationship of some functional polymorphisms in pro-inflammatory cytokine genes with the significant increase in the susceptibility to several CVD, abdominal aortic aneurysms included. Consistent with this, a prospective multicenter observational open-label cohort study of patients, the MA3RS study [20], has demonstrated the importance of monitoring aortic wall inflammation via ultrasmall superparamagnetic particles of iron oxide-enhanced magnetic resonance imaging for significantly predicting both the rate of aneurysm growth, and the risk of severe complications, such as aneurysm rupture [20].

**Figure 1:**
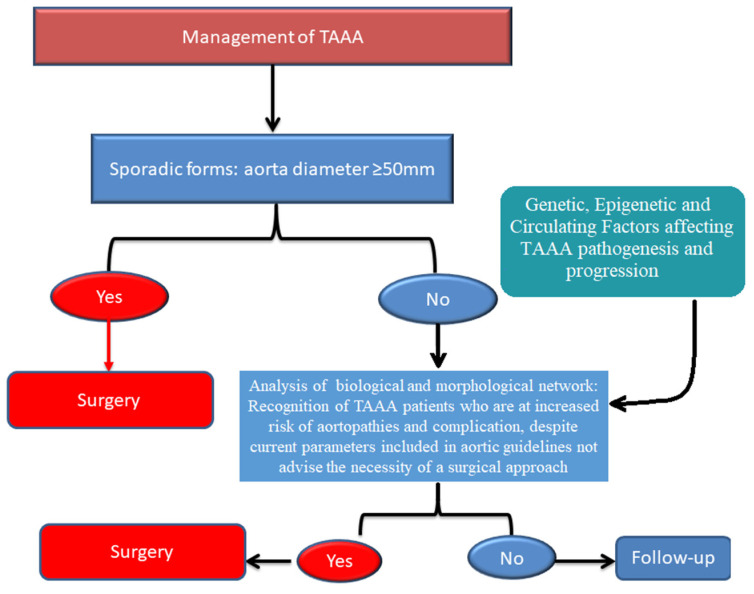
Management of patients with TAAA (Flow Chart). This algorithm was proposed to include biological and morphological network of genetic, epigenetic and circulating markers useful for updating current guidelines on the TAAA management behind the aortic diameter [22].

These interesting data have prompted evaluation of the contribution of inflammatory pathways and cytokines to risk and pathogenesis of sporadic thoracic ascending aortic aneurysms (TAAA). Our group has recently demonstrated the crucial role of TLR-4/NF-kβ and TGF-β in the risk and progression of TAAA and type A dissection and evidenced genetic variants in their genes as important risk determinants of these diseases [8,9,21,22,23]. The group of researchers involved in Genetically Triggered Thoracic Aortic Conditions (GenTAC) registry study, has also found a significant association between the high systemic levels of *IL-6* and aortic dimensions in patients with aortopathies [24]. Another group has assessed an overexpression of *IL-1β* in patients with TAAA [25]. In this view, the identification of genetic, epigenetic and circulating biomarkers might be of help for update the current TAAA guidelines on the surgical timing, which is the key for positively influencing the survival of affected patients and is based exclusively on aorta diameter [22]. A growing body of evidence indicates that a biological and morphological network of risk factors might be considered and aortic ruptures and dissections might also take place in ascending aortas having smaller sizes than those recommended by the current guidelines [22] (Figure 1).

In line with these recent data, and for validating the associations previously observed [22], here, we aimed to investigate the potential association of functional polymorphisms in *IL-6,* *IL-1A, IL-1B, IL-18,* and *Tumor necrosis factor (TNF)A* genes with TAAA susceptibility, pathogenesis, and outcome, in 144 patients affected by sporadic TAAA and 150 age/gender matched controls. Five functional promoter polymorphisms in the genes encoding for *IL-6*, *IL-1α*, *IL-1β*, *IL-18* and *TNF-α* pro-inflammatory cytokines, known to be functionally important, because they influence both the transcription rate of the related genes and cytokine plasma concentrations, have been selected [26,27,28,29,30]. In addition, we evaluated the eventual genotypes of these gene variants able to influence in a significant manner the systemic levels of the analyzed cytokines and other inflammatory markers (i.e., proteases), as well as the amounts of tissue infiltrated CD3+CD4+CD8+CD68+CD20+ immune/inflammatory cells. Correlations of the polymorphisms (i.e., their genotypes) with molecular and cellular aorta wall impairment, leukocyte telomere length attrition and telomerase activity alterations, able to suggest the rate of tissue biological aging, tissue chronic inflammation, related damage, and the resulting aorta remodeling/degeneration, reflecting the aneurysm growth and/or rupture, were also estimated.

## 2. Materials and Methods

### 2.1. Patients and Controls

Our study included a population of 144 subjects with TAAA (101 men and 43 women; mean age: 70.3 ± 2.6 years) and 150 age/gender matched healthy subjects (100 men and 50 women; mean age: 69.5 ± 1.6 years), as shown in Table 1. Patients were recruited from February 2017 to December 2017, in the Units of Cardiac Surgery and Cardiology (Department of Cardiac Surgery, University of Rome ‘Tor Vergata’, Rome, Italy). Exclusion criteria [31,32,33,34]: (a) cardiovascular diseases; (b) genetic, familial and sporadic connective tissue disorders; (c) congenital aorta valve diseases, such as bicuspid valve syndrome; (d) infectious and inflammatory diseases. A total of 150 healthy age and gender matched controls were recruited after clinical and laboratory evaluation.

Table 1 reports clinical and demographic data (including comorbidities) obtained from patients’ medical records.

### 2.2. DNA Samples and Genotyping

DNA samples from patients and controls, extracted from peripheral blood and purified by using QIAamp Blood DNA Maxi kit (Qiagen, Dusseldorf, Germany), were typed for five polymorphisms located in the promoter region of the five selected candidate genes codifying pro-inflammatory cytokine reported in Table 2.

The allelic and genotypic frequencies of these gene variants were detected using Kaspar assay on demand developed by KBioscience Ltd. (KBioscience, Middlesex, UK) and based on a homogeneous Fluorescence Resonance Energy Transfer (FRET) detection and allele specific PCR routinely used in our laboratory [21].

### 2.3. Quantifications of Systemic Levels of IL-1β, IL-6, MMP-9 and MMP-2

Plasma levels of inflammatory cytokines (*IL-1β*, and *-6*), and MMP-9 and -2 were measured by ELISA and commercial kits (R&D Systems, Minneapolis, MN, USA), according to the manufacturer’s instructions. Detection limits were 0.7 pg/mL, 0.5 pg/mL, 0.154 ng/mL, 0.156 ng/mL for *IL-1β*, *IL-6*, *MMP-9* and *-2*, respectively. All assays were run in duplicate.

### 2.4. Aortic Specimens and Histopathological Assays and Apoptosis Evaluation

Full aortic segments with resected normal as well as aneurysmatic aortic wall from tubular-ascending aorta were collected from all patients with TAAA. They were microscopically examined, after staining (hematoxylin-eosin, Weigert, van Gieson and Alcian-PAS staining, see Figures S1 and S2 in Supplementary Materials) according to the 2016 consensus criteria for aorta histology [35].

We also assessed apoptosis by perform TdT (Terminal deoxynucleotidyl Transferase)-mediated X-dUTP (deoxyuridine triphosphate nucleotides) nick end-labeling (TUNEL) reaction (Roche Diagnostics S.p.A, Milano, Italy) on deparaffined sections of full-thickness aortic wall (5 µm), as previously described [31,32,33,34,36].

### 2.5. Immunohistochemical Assays

Immunohistochemical analyses were performed on 5 μm-thick paraffin-embedded sections incubated for 1 h with appropriate dilutions of specific monoclonal antibodies (Ab)s against CD3 (Clone LN10, NCL-L-CD3, clone PS1, Novocastra Laboratories Ltd., Newcastle upon Tyne, UK, 1:100), CD45 (Santa Cruz, Biotechnology, Inc, Santa Cruz, CA, USA, 1:100), CD4 Ab-2 (Clone 1 F6, NeoMarkers, Inc, Fremont, CA, USA, 1:10), CD8 Ab-1 (Clone C8/144B, NCL-L-CD8 295 mouse Novocastra Laboratories Ldt, Newcastle upon Tyne, UK, 1:50), CD20 (clone L 26, Dako Italia Spa, Milan, Italy, 1:50)), CD68 (clone PG-M1, Dako Italia SpA, Milan, Italy, 1:50), MMP-9 (Clone 15W2, NCL-MMP9 439, Novocastra Laboratories Ltd., Newcastle upon Tyne, UK, 1:50), or isotype-matched controls. After washing in TBS 1X (Tris-buffered solution), staining was performed by biotinylated antibodies and streptavidin labeled with peroxidase (Dako, North America, Inc, Via Real Carpinteria, CA, USA) and was detected using 3-amino-9-ethylcarbazole substrate (AEC). Counterstaining of cells and tissue sections was performed using aqueous hematoxylin (Novocastra Laboratories Ldt, Newcastle upon Tyne, UK). Inflammatory and immune cells were counted in 10 contiguous high-power fields (magnification 400×) under an Olympus fluorescent microscope (Olympus America Inc, Center Valley, PA, USA) by two independent observers.

### 2.6. Semi-Quantitative Evaluation of MMP-9 by Immunohistochemical Assays

A semi-quantitative evaluation of MMP-9 amount in aortic specimens was performed. Staining was classified as low, moderate, or high amount (see Figure S2).

### 2.7. Telomere Length Assay and Telomerase Activity Evaluation

The mean terminal restriction fragment (TRF) length, was detected by chemiluminescence technique and using the TeloTAGGG telomere length assay kit (Roche Diagnostics, Indianapolis, IN, USA), according to the manufacturer’s instructions. The mean terminal restriction fragment (TRF) was calculated applying the following formula: TRF = (∑(*i*OD))/(∑(*i*OD/*Li*)), based on measurement of is the optical density at a given position on the gel (OD*_i_)* and the position corresponding molecular weight (*L_i_*). As previously described [36], the mean TRF of electrophoresis runs from cases and controls were adjusted to the standardized internal control.

For quantitative analysis of telomerase activity, a Telomeric Repeat Amplification Protocol (TRAP) [36] and a photometric enzyme immunoassay were performed using Telo*TAGGG* Telomerase PCR Elisa ^Plus^ kit (Roche Diagnostics, Indianapolis, IN, USA), according to the manufacture’s protocol [36].

### 2.8. Statistical Analysis

Significant differences in frequencies between the two groups were calculated using the χ2 test and appropriate tables (2 × 2, 3 × 2). Unpaired *t*-test (Welch corrected) was utilized to analyze the quantitative data between two groups whereas one-way ANOVA or Kruskal–Wallis test followed by Bonferroni correction was applied to compare more than two groups. The correlations between two continuous variables were assessed with Pearson’s test, or non-parametrical Spearman correlation test. Multiple logistic regression analyses of dominant and recessive models were applied to patient group compared with control group. Odds ratios (OR), 95% confidence intervals (95% C.I.), and *p* values were determined. SPSS software version 20 (SPSS Inc., Chicago, IL, USA) was used. Differences were considered significant when a *p* value < 0.05 was obtained by comparison between the different groups.

## 3. Results

### 3.1. Frequencies and Associations of Cytokine Functional Promoter Polymorphisms with TAAA Risk in Enrolled Groups

Genotype frequencies of all studied population fit to Hardy–Weinberg equilibrium. The comparison of genotype distributions and allele frequencies between cases and controls allows to detected only significant differences both in genotype distributions and allele frequencies of the rs.16944 *IL-1B* and rs.1800795 *IL-6* gene variants, between the cases and the matched controls (Table 3).

In particular, we observed that the TAAA cases showed a significant frequency (0.153 vs. 0.02 and *p* < 0.00009; 0.152 vs. 0.034 and *p* < 0.0015, by Chi-square test) of TT and CC genotypes, respectively of the rs.16944 *IL-1β* and rs.1800795 *IL-6* gene variants than the controls. Analogously, the frequency of T and C alleles, respectively of the rs.16944 *IL-1β* and rs.1800795 *IL-6* gene variants, was significantly higher in cases than controls (0.236 vs. 0.087 and *p* < 0.000001; 0.222 vs. 0.100, and *p* < 0.00008 by Chi-square test).

Furthermore, the logistic regression analyses of dominant and recessive models performed between TAAA cases and controls showed a significant enrichment of the recessive genotypes of two polymorphisms in cases respect to controls, associated with significant risk TAAA values (Table 4).

### 3.2. Higher Systemic Plasma Levels of Inflammatory Systemic Molecules and Aorta Tissue Immune/Inflammatory Cells in Cases with the Combined -511T IL-1B/-174C IL-6 Genotype than Controls Bearing the Same Genotype

**Figure 2:**
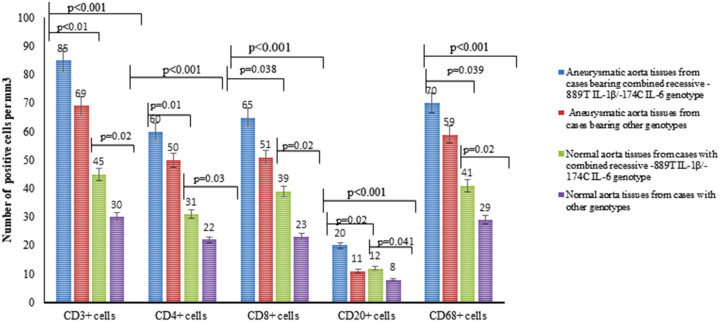
Immunohystochemical quantification of lymphocytes, T cell subpopulations, and macrophages in aorta samples (aneurysmal and normal areas) from cases with combined -511T *IL-1B*/-174C *IL-6* genotype vs. cases with other genotypes. CD3, CD4, CD8, CD20, and CD68 positive cells in media and adventitia and in 10 contiguous high-power fields (magnification 400×) were counted by two independent observers. Significant increased amounts of CD3+CD4+CD8+CD68+CD20+ cells were observed by comparing their values (medium values± SD) among the four groups and the two groups (by ANOVA and *t* test). Surprisingly, cases with the combined genotype had higher numbers of these cells than cases with other genotypes, in both aneurysmatic and normal aorta tissue areas.

The significant enrichment in the frequencies of rs.16944 *IL-1B* and rs.1800795 *IL-6* gene variants in TAAA cases than controls and the high OR values of recessive genotypes in the cases led us to suppose that these polymorphisms might have the role of genetic modifiers, promoting TAAA pathogenesis and outcome. Consequently, we examined the biological effects mediated by the rs.16944 *IL-1B* and rs. 1800795 *IL-6* gene variants in cases and controls bearer or not of *-511T IL-1B/-174C IL-6* combined genotypes, by quantifying both the amount of aorta tissue cellular infiltration of CD3+CD4+CD8+CD68+CD20+ inflammatory immune cells (Figure 2) and the plasma levels of the two *IL-1β* and *IL-6* cytokines and metalloproteinases (MMP)-2 and -9 (Table 5).

The comparisons effectuated between the cases and controls bearer or not of *-511T IL-1B/ -174C IL-6* combined genotypes demonstrated that the cases positive for combined genotype had significantly higher levels of systemic inflammatory cytokines and proteases than the controls with the same genotype or carriers of other genotypes (Table 5). Besides, cases carrying *-511T IL-1β/-174C IL-6* genotype had significantly higher amount of tissue CD3+CD4+CD8+CD68+CD20+ inflammatory immune cells both in normal and aneurysmatic aorta tissues (Figure 2).

Overall, these results also led us to suppose that the rs.16944 *IL-1B* and rs.1800795 *IL-6* gene variants might also be considered not only genetic modifiers of TAAA pathogenesis, but also potential predictive and prognostic TAAA biomarkers because they correlated with the raise and grade of both systemic and tissue chronic inflammation, remodeling and degeneration when they are both present.

### 3.3. A Significant Impairment of the Leukocyte Telomere Length and Telomerase Activity in Cases with Combined the -511T IL-1B/-174C IL-6 Genotype than Controls

To assess the predictive and prognostic risk effects of combined -*511T IL-1B/-174C IL-6* genotype in cases than controls, we evaluated the eventual telomere/telomerase system’s impairment, it being the gold standard biomarker for evaluation of the grade of biological aging of all the tissues. As widely reported in our recent work [36] and documented in the literature (see the relevant data of Wilson and colleagues, [37]), it reflects in an exact manner both the grade of chronic inflammation and remodeling/degeneration of all the body tissues, or, better, the biological age of all the tissues.

Therefore, we examined the mean of blood leukocyte telomere length and telomerase activity, using terminal restriction fragment assay (TRF test,), and a relative quantitative analysis in according to Telomeric Repeat Amplification Protocol (TRAP).

As reported in Table 6, we have detected a significant impairment of telomere/telomerase leukocyte system in TAAA cases with combined recessive *-511T IL-1B/-174C IL-6* genotype than controls with the same genotype. In addition, controls positive for the combined genotype had impairment of telomere/telomerase leukocyte system than other control individuals, carriers of another genotype. This finding might suggest that the combined recessive -*511T IL-1B/-174C IL-6* genotype is associated with a significant risk of early biological aging of cardiovascular system, and consequently to develop an early aorta remodeling and degeneration, that might evolve in TAAA onset.

### 3.4. Univariate Analysis of Combined -511T IL-1B/-174C IL-6 Genotype with Increase in MMP9 Amount, Elastic Fragmentation, Medial Cell Apoptosis, Cystic Medial Changes in Hystological Speciments

To test whether the combined *-511T IL-1β/-174C IL-6* genotype correlated with aorta tissue remodeling/degeneration, elastic fragmentation, tissue aorta MMP-9 amount, medial cell apoptosis, cystic medial changes in TAAA cases were detected and a univariate regression analysis was applied. As reported in Table 7, a significant correlation was detected between the combined genotype and aorta alterations, including an increased MMP-9 amount, elastic fragmentation, medial cell apoptosis and cystic medial changes. Besides, it significantly correlated with the augment of aorta diameter (r = 0.15 and *p* = 0.01, by linear Pearson correlation test; data not shown) in the cases bearer.

## 4. Discussion

Inflammation plays a crucial role in the CVD development and progression. Accordingly, our and other groups have demonstrated significant associations between many inflammatory pathways, and mediators, and TAAA [8,9,21,22,23,24,25]. Pro-inflammatory cytokines, and particularly IL-6, have been shown to represent significant drivers of CVD [38,39], aneurysms included. Its crucial role in CVD has been proven by prospective studies, demonstrating how high basal plasmatic levels of IL-6, having pro-inflammatory and procoagulant effects, are potent CVD risk factors [40,41,42,43]. In addition, other pieces of evidence arrive from the significant results of a recent meta-analysis of 74 studies, showing a significant association of the -174C allele (rs1800795) of *IL-6* gene polymorphism with several CVD, including myocardial infarction, coronary artery disease, ischemic stroke, peripheral arterial occlusive disease, and heart failure [16], in spite of functional effect of this allele on IL-6 serum level [44]. Recent studies have also confirmed its role in abdominal aortic aneurysms [42,43], Here, we demonstrated the role of *IL-6* and *IL-1B* in TAAA disease. Precisely, we evidenced that the functional -174G>C (rs1800795) and -511C>T (rs16944) polymorphisms, respectively in *IL-6* and *IL-1B* genes, were significantly associated with TAAA risk. Such gene variants increased the TAAA susceptibility under allelic (C and T, respectively), homozygous (CC and TT) and heterozygous (GC and CT) genotypes. Higher systemic levels of the two cytokines were significantly assessed in plasma samples of cases bearing such gene variants than controls, and particularly when carriers of combined recessive *-511T IL-1B/-174C IL-6* genotype. Likewise, cases with the combined recessive *-511TIL-1B/-174C IL-6* genotype showed higher systemic plasma levels of MMP-9 and-2 respect to controls. This appears to suggest that the two polymorphisms induce a higher systemic inflammatory pressure, that might likely result in a more marked cytokine-induced aorta remodeling and degeneration. Accordingly, we detected in aorta tissues from the cases with combined recessive *-511T IL-1B/-174C IL-6* genotype, and particularly in aneurisma vs. normal regions, a significant immune/inflammatory cellular infiltration. Aneurisma tissues are characterized by the presence not only of a significant number of CD68+ monocyte/macrophage cells, but also by a high quantity of the T CD4+ and CD8+ cells followed by a reduced number of B lymphocytes with the typical CD20+ marker. Generally, their presence indicates a chronic inflammation response [45,46]. However, the phenotypes of the T CD4+, CD8+, and B cell subpopulations were not analyzed in our study, as well as gene expression and cytokine production. In fact, it has recently been reported that the cytokines produced by CD4+ and CD8 + T-lymphocytes and their subsets, such as CD248+ CD8+ T cells [47], play a causative role in the initiation and progression, as well as in the suppression, of aorta remodeling associated with onset of pathological conditions, such as aorta dilatation and aneurysm development [46,48]. Of note, for example, is the role of the abovementioned CD248+ CD8 + T [47] cells subset and its cytokine profile, that has been recently demonstrated to suppress the pathological vascular remodeling in human TAAA. This underlines the imperative necessity to investigate the type of T subsets in the aneurysm aorta tissues and their cytokine profile.

In this view these results allow to suggest that the rs16944 *IL-1B* and rs.1800795 *IL-6* gene variants might be considered not only genetic modifiers of TAAA pathogenesis, but also potential predictive and prognostic TAAA biomarkers. Consistent with these considerations, the rs16944 *IL-1B* and rs1800795 *IL-6* gene variants correlated with the rise and grade of both systemic and tissue chronic inflammation and biological aging of aorta tissue. Accordingly, cases with the combined *-511T IL-1B/-174C IL-6* genotype than control carriers of the same genotype had shorter telomeres and a reduced telomerase activity. Leukocyte telomere length and telomerase activity are strictly associated with the grade of biological aging of body tissues, organs and systems, and reflect both the grade of tissue chronic inflammation and remodeling/degeneration [36,49,50,51]. The significant associations between the impairment of telomere length and telomerase activity and -174G>C (rs1800795) *IL-6* and -511C>T (rs16944) *IL-1B* gene polymorphisms, seem to confirm the contribution of combined genotype in TAA onset and progression and, consequently, it might represent a TAAA predictive and prognostic risk marker. An additional confirmation arrives from the significant correlations between the combined *-511T IL-1B/-174C IL-6* genotype and the TAAA tissue aorta typical alterations, including elastic fragmentation, MMP-9 aorta tissue amount, medial cell apoptosis and cystic medial changes.

## 5. Conclusions

Taken together, the results obtained appear to strongly suggest that the two -174G>C (rs1800795) *IL-6* and -511C>T (rs16944) *IL-1B* gene polymorphisms are genetic risk factors for TAAA, in accordance with the literature data reported on the several CVD, as mentioned above [38,39,40,41,42,43]. However, to the best of our knowledge, this study represents the first performed in TAAA and, consequently, confirmations on large numbers of individuals enrolled are imperative.

**Figure 3:**
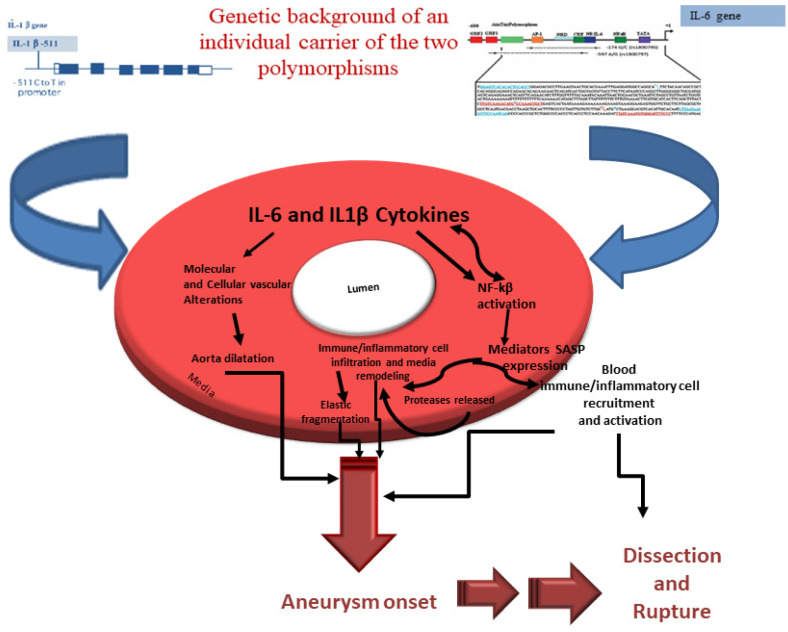
Our model on the role of rs1800795 *IL-6* and rs16944 *IL-1B* gene polymorphisms in TAAA onset. Subjects carrying combined *-511T IL-1B/-174C IL-6* genotype show high significant levels of two related cytokines, known have a key role in the onset and progression of CVD. In the context of TAAA pathogenesis, they mediate cellular and molecular alterations of intima, inducing endothelium dysfunction and, in turn, immune/inflammatory infiltration, and media remodeling/degeneration. Such determines aorta dilation and, consequently, aneurysm onset and its complications, dissection, and rupture.

The crucial genetic mechanisms related to higher susceptibility to the same diseases may be realized by different genotypes, which can affect different mediating mechanisms, in populations of different genetic backgrounds [52,53,54]. In our case, this must be considered in relation to the biological effects mediated by the two -174G>C (rs1800795) *IL-6* and -511C>T (rs16944) *IL-1B* gene polymorphisms, and particularly by the combined *-511T IL-1B/-174C IL-6* genotype that might contribute to the different steps of TAAA pathogenesis and progression (Figure 3). Afterward, we explored this issue considering the ethnicity of our population in study. Being a Caucasian population, the literature data have reported a significant association only under homozygous recessive model (TT and CC) of the polymorphisms examined in our study, and particularly for -174G>C (rs1800795) *IL-6* polymorphism [16].

Some limitations also characterize our study. First, we need to consider the sample size. However, the total number of subjects enrolled was relatively small given the incidence and prevalence of TAAA, even if the validation of our data needs to be confirmed in a larger sample of patients and controls. Second, our study was an association analysis that correlated genotype to TAAA risk, cytokine levels and histologic, immune-histochemical, telomere/telomerase markers of arterial wall degeneration, molecular pathways of arterial damages should be further investigated, as well as the tissue molecule expression. Third, the biological effects of the two polymorphisms studied are complex and other genetic variants of their functional pathways might be implied [55]. Consequently, further research is needed to fully understand the contribution of two -174G>C (rs1800795) *IL-6* and -511C>T (rs16944) *IL-1B* gene polymorphisms in TAAA.

However, genotyping could consent to identify TAAA risk individuals and might represent a helpful biomarker useful in drive preventive treatments for delaying or stopping onset and progression of TAAA. Specific inhibitors of inflammation (nonsteroidal anti-inflammatory drugs or other more sophisticated preventive approaches (i.e., agonists of IL- receptors or anti-*IL-6* or *IL-1β* antibodies), might be used, as well as more appropriate surgical approaches and decisions in subjects positive for this genotype.

## Figures and Tables

**Table 1:** Demographic and clinical characteristics of population enrolled.

Variables	TAAA Cases	Controls	*p* ^1^
	*n* = 144	*n* = 150	
***Demographic characteristics***			
Age, mean (SD)	70.3 (2.6)	69.5 (1.6)	n.s.
Male sex, No. (%)	101 (70.1)	100 (66.6)	n.s.
Female sex, No. (%)	43 (29.9)	50 (33.4)	n.s.
Body mass index, mean (SD)	25 (4.3)	24.8 (3.1)	n.s.
***Size and location of TAAA***			
Size (mm), mean (DS)	52.6 (7.1)	-	-
Localization N. (%): Tubular ascending aorta	144 (100)	-	-
***Comorbidity conditions, No (%)***			
CVD Family History	18 (12.5)	10 (6.7)	n.s.
Smoking	50 (34.7)	50 (33.4)	n.s.
Hypertension	75 (52)	60 (40)	0.03
Dyslipidemia	22 (15.3)	12 (8)	0.05
Diabetes mellitus	13 (9)	8 (5.3)	n.s.
Renal failure	0 (0)	0 (0)	n.s.
Dissection	0 (0)	0 (0)	n.s.
***Coronary syndrome* No (%)**	2 (0)	0 (0)	n.s.

^1^ *p* was determined by *t* test for quantitative variables, or Pearson χ2 test for qualitative variables.

**Table 2:** Genes and SNPs (accession number from https://www.ncbi.nlm.nih.gov/snp/?term, accessed on 21 April 2021) investigated in the study [24,25,26,27,28].

Genes	SNPs	Chr Localization	Position	Alleles	Biological Effects
*IL-1A*	rs1800587	2:112785383	−889	C>T	*Increased levels of gene transcription*
*IL-1B*	rs16944	2:112837290	−511	C>T	*Increased levels of gene transcription*
*IL-6*	rs1800795	7:22727026	−174	G>C	*Variable levels of gene transcription*
*IL-18*	rs187238	11:112164265	−137	G>C	*Increased levels of gene transcription*
*TNFA*	rs1800629	22:23894205	−308	G>A	*Increased levels of gene transcription*

**Table 3:** Genotype distributions and allele frequencies of gene variants (SNPs) typed in case and control groups. All genotypes were in Hardy–Weinberg equilibrium.

*Candidate Gene*	SNP Reference Number	Alleles				*p* Value * (2 × 2 Table)	Genotypes						*p* Value * (3 × 2 Table)
*IL-1A*	rs1800587 (-889C/T)	C		T			CC		CT		TT		
		N	F	N	F		N	F	N	F	N	F	
	**Cases**	193	0.670	95	0.330	**n.s.**	61	0.424	70	0.486	13	0.090	**n.s.**
	**Controls**	209	0.697	91	0.303		72	0.480	65	0.433	13	0.087	
*IL-1B*	rs16944 (-511C>T)	C		T			CC		CT		TT		
		N	F	N	F		N	F	N	F	N	F	
	**Cases**	220	0.764	68	0.236	**0.000001**	98	0.680	24	0.167	22	0.153	**0.00009**
	**Controls**	274	0.913	26	0.087		127	0.847	20	0.133	3	0.02	
*IL-6*	rs1800795 (-174G>C)	G		C			GG		GC		CC		
		N	F	N	F		N	F	N	F	N	F	
	**Cases**	224	0.778	64	0.222	**0.00008**	102	0.708	20	0.140	22	0.152	**0.0015**
	**Controls**	270	0.900	30	0.100		125	0.833	20	0.133	5	0.034	
*IL-18*	rs187238 (-137G/C)	G		C			GG		GC		CC		
		N	F	N	F		N	F	N	F	N	F	
	**Cases**	205	0.712	85	0.288	**n.s.**	67	0.465	70	0.486	7	0.049	**n.s.**
	**Controls**	219	0.730	81	0.270		82	0.547	55	0.367	13	0.087	
*TNFA*	rs1800629 (-308G/A)	G		A			GG		GA		AA		
		N	F	N	F		N	F	N	F	N	F	
	**Cases**	251	0.872	37	0.128	**n.s.**	112	0.777	27	0.188	5	0.035	**n.s.**
	**Controls**	270	0.900	30	0.100		124	0.827	22	0.147	4	0.026	

N = number; F = frequency (genotype distribution and allele frequencies). * *p* value was calculated by using χ2 test and appropriate 3 × 2 and 2 × 2 tables.

**Table 4:** Multiple logistic regression analyses of dominant and recessive models applied to patient group compared with control group.

SNP	Model	Numbers	OR (95% C.I.)	*p* Values *
rs16944 (-511C>T)	Dominant CC + CT/TT	Cases: 122/22 Controls: 147/3	0.11 (0.03–0.38)	<0.00001
	Recessive CT + TT/CC	Cases: 46/98 Controls: 23/127	2.59 (1.47–4.56)	0.0006
rs1800795 (-174G>C)	Dominant GG + GC/CC	Cases: 122/22 Controls: 145/5	0.21 (0.07–0.57)	0.0008
	Recessive CC + GC/GG	Controls: 25/125 Cases: 42/102	2.05 (1.17–3.06)	0.0077

* *p* value was calculated by using χ2 test and appropriate 3 × 2 and 2 × 2 tables; OR was calculated with Fisher’s exact test.

**Table 5:** Plasmatic levels (average ± standard deviation) of *IL-6* and *IL-1β* cytokines and metalloproteases in the 144 TAAA patients and 150 age/gender matched controls, stratified according the presence of combined recessive (-511T *IL-1B/-174C IL-6*) genotype.

Plasmatic Protein	-511T *IL-1B/-174C IL-6* Positive Patients (*n* = 56)	-511T *IL-1B/-174C IL-6* Negative Patients (*n* = 88)	-511T *IL-1B/-174C IL-6* Positive Controls (*n* = 23)	-511T *IL-1B/-174C IL-6* Negative Controls (*n* = 127)	*p* Values *		
					-511T *IL-1B/-174C IL-6* Positive Patients vs. Positive Controls	-511T *IL-1B/-174C IL-6* Positive vs. Negative Patients	-511T *IL-1B/-174C IL-6* Positive vs. Negative Controls
IL-6 (pg/mL)	22 ± 2.1	16 ± 0.9	9 ± 5.6	6.8 ± 2.1	<0.0001	0.01	0.03
IL1-β (pg/mL)	23 ± 2	15 ± 2.2	15 ± 6	9 ± 2.8	<0.01	0.01	<0.01
MMP-2 (ng/mL)	63 ± 4.5	52 ± 2.3	22 ± 5.6	14 ± 5.3	<0.0001	0.01	<0.001
MMP-9 (ng/mL)	60 ± 3.1	51 ± 3.1	23 ± 6.1	15 ± 2.6	<0.0001	0.01	<0.001

* *p* was determined by t test with Welch correction.

**Table 6:** Mean TRF length, mean values of relative telomerase activity (RTA), in leukocytes from patients and controls positive or negative for combined genotype ^1^.

Evaluations	Case’s Carriers (*n* = 56)	Control’s Carriers (*n* = 23)	Cases with Other Genotypes (*n* = 88)	Controls with Other Genotypes (*n* = 127)	*p*1 * Values	*p*2 ** Values	*p*3 *** Values	*p*4 **** Values
Mean TRF length	4899 ± 0.569 bp	6588 ± 0.449 bp	5680 ± 0.176bp	7500 ± 0.656 bp	<0.001	<0.0001	0.01	<0.0001
Mean RTA values	12.6 ± 3.2	60.8 ± 5.6	27 ± 3.3	75 ± 6.2	<0.0001	<0.0001	0.001	<0.0001

^1.^ unpaired t test with Welch correction was used for statistical analyses. Data are reported as mean ± standard deviation. * *p*1 values obtained comparing patients with combined genotype vs. controls with combined genotype; ** *p*2 values obtained comparing patients with combined genotype vs. controls with other genotypes; *** *p*3 values obtained comparing patients with combined genotype vs. patients with other genotypes; **** *p*4 values obtained comparing controls with combined genotype vs. controls with other genotypes.

**Table 7:** Univariate correlations between elevation of MMP9 amount, elastic fragmentation, medial cell apoptosis, cystic medial changes, and the combined recessive (-511T *IL-1B/-174C IL-6*) genotype in cases.

Variables	r Values	*p* Values *
Elastic fragmentation	0.21	0.001
Elevation of MMP 9 amount	0.17	0.02
Medial cell apoptosis	0.16	0.001
Cystic medial changes	0.18	0.04

* linear Pearson correlation test, or non-parametric Spearman correlation test, when appropriate.

## Data Availability

All data generated or analyzed during this study are included in this published article.

## Supplementary Materials

The following are available online at https://www.mdpi.com/article/10.3390/biom11070943/s1: Aortic specimens and histopathological assays and apoptosis evaluation, Figure S1: Control aortas and histo-pathological abnormalities in aorta tissues of S-TAA patients; Figure S2: Medial apoptosis and MMP-9 amount in tissue samples.
