# Supplementary material for: Polymorphisms of Pro-Inflammatory IL-6 and IL-1β Cytokines in Ascending Aortic Aneurysms as Genetic Modifiers and Predictive and Prognostic Biomarkers
Source: Biomolecules. 2021 Jun 25;11(7):943. doi: 10.3390/biom11070943 (PMC8301826; doi:10.3390/biom11070943)
Supplement: Supplementary file 1 [file biomolecules-11-00943-s001.zip › Aortic specimens and histopathological assays and apoptosis evaluation.pdf]

Aortic specimens and histopathological assays and apoptosis evaluation. Full aortic segments with resected normal as well as aneurysmatic aortic wall from tubular-ascending aorta were collected from all patients with TAAA.They were fixed in 10% neutral buffered formalin for 24 hours and then processed for routine paraffin embedding. Surgical specimens subsequently were photographer and measured (maximum transverse diameter). For microscopic examinations, multiple histological sections from each sample were prepared and stained (with hematoxylin-eosin, Weigert-van Gieson, Alcian-PAS; see Fig. 1S) according to the 2016 consensus criteria for aorta histology [35]. We also assessed apoptosis by performed TdT (Terminal deoxynucleotidyl Transferase)-mediated X-dUTP (deoxyuridine triphosphate nucleotides) nick end-labeling (TUNEL) reaction ("In situ cell death detection kit", Roche Diagnostics S.p.A, Milano, Italy) on full-thickness aortic wall paraffin sections (5 µm), as previously described in our previous papers (see Fig. 2S). [31-34, 36].

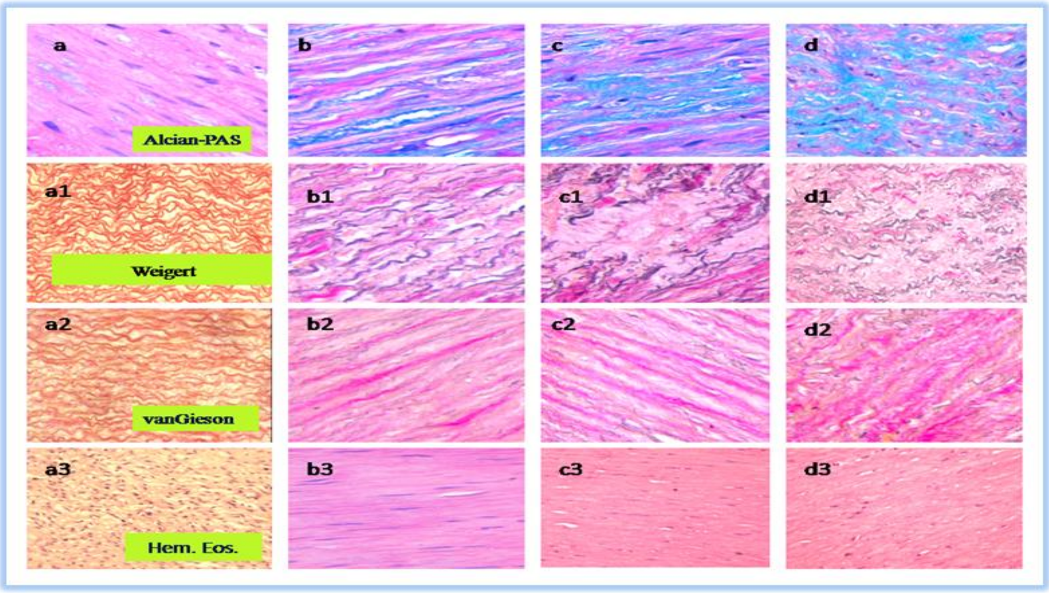

Fig. 1S. Control aortas and histo-pathological abnormalities in aorta tissues of S-TAA patients.
Normal aorta (a, a1, a2, a3). Cystic medial changes of grade I (b), II (c) and III (d); Elastic fragmentation of grade I (b1), II (c1) and III (d1). Medial fibrosis of grade I (b2), II (c2) and III (d2). Medial necrosis of grade I (b3), II (c3) and III (d3).

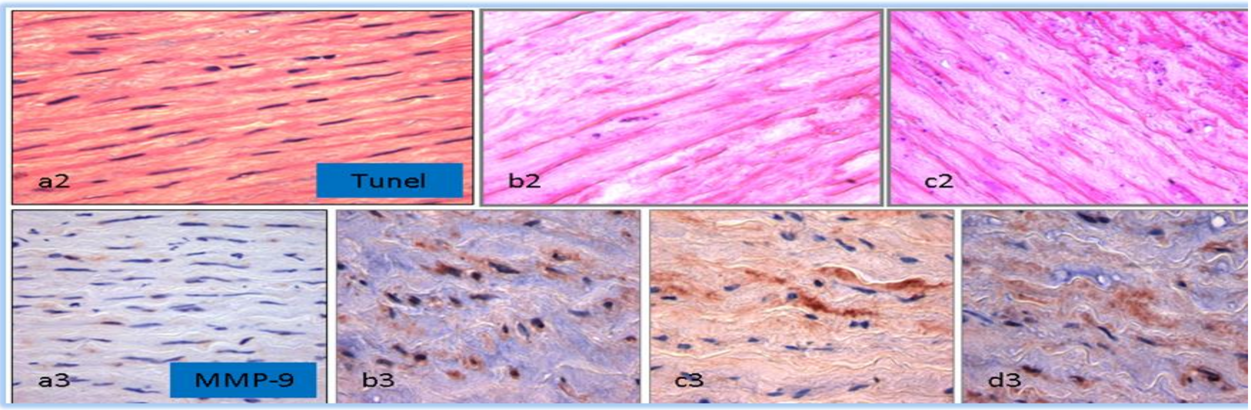

Fig.2S Medial apoptosis and MMP-9 amounts in tissue samples. In a2 and a3 images of control aorta wa[illegible] conditions. In b2 and c2 images of focal and plurifocal medial apoptosis, respectively, in patient tissues. In [illegible] (low), c3 (moderate) and d3 (elevated) levels of MMP-9 in patient samples.
