# Supplementary figures and images for: Polymorphisms of Pro-Inflammatory IL-6 and IL-1β Cytokines in Ascending Aortic Aneurysms as Genetic Modifiers and Predictive and Prognostic Biomarkers
Source: Biomolecules. 2021 Jun 25;11(7):943. doi: 10.3390/biom11070943 (PMC8301826; doi:10.3390/biom11070943)

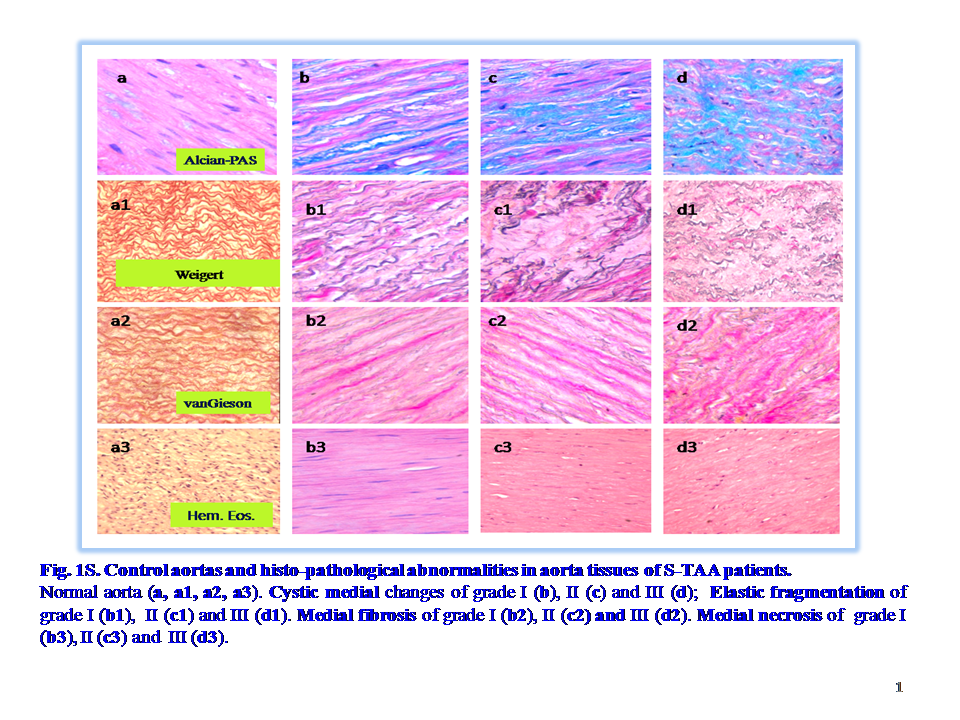

Supplement: Supplementary file 1 [file biomolecules-11-00943-s001.zip › Figure S1.tif]

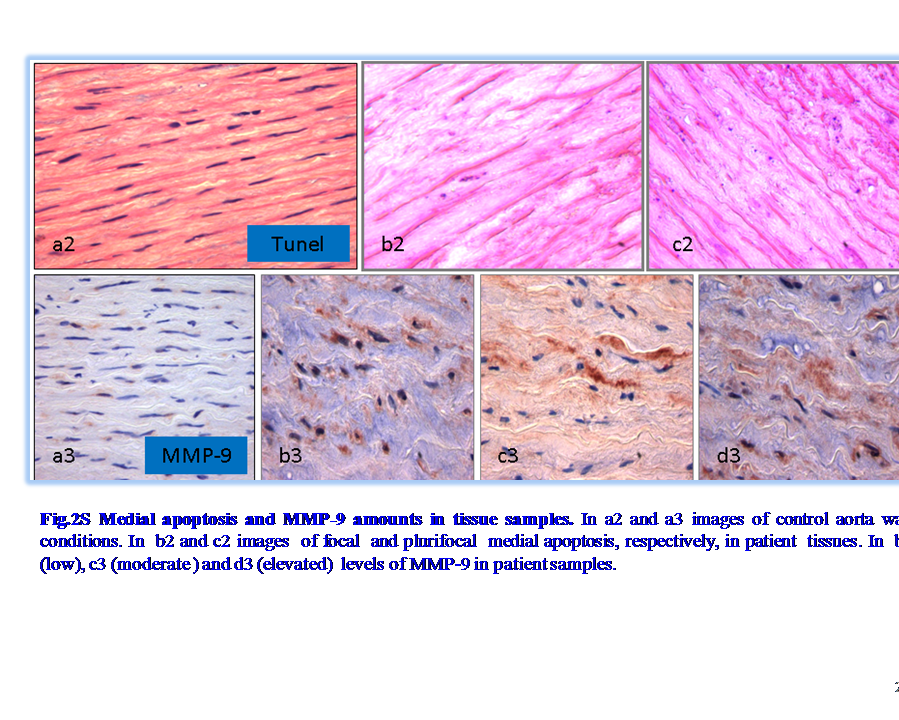

Supplement: Supplementary file 1 [file biomolecules-11-00943-s001.zip › Figure S2.tif]
